# The Effect of Elevated Curing Temperatures on High Ye’elimite Calcium Sulfoaluminate Cement Mortars

**DOI:** 10.3390/ma12071072

**Published:** 2019-04-01

**Authors:** Yeonung Jeong, Craig W. Hargis, Hyunuk Kang, Sung-Chul Chun, Juhyuk Moon

**Affiliations:** 1 Construction Technology Research Center, Korea Conformity Laboratories (KCL), 199, Gasan Digital 1-ro, Geumcheon-gu, Seoul 08503, Korea; yeonungjeong@kcl.re.kr; 2 Department of Construction Management, University of North Florida, 1 UNF Dr., Jacksonville, FL 32224, USA; craig.hargis@unf.edu; 3 Department of Civil and Environmental Engineering, Seoul National University, 1 Gwanak-ro, Gwanak-gu, Seoul 08826, Korea; kanghu93@snu.ac.kr; 4 Division of Architecture and Urban Design, Incheon National University, Incheon 22012, Korea; 5 Institute of Construction and Environmental Engineering, 1 Gwanak-ro, Gwanak-gu, Seoul 08826, Korea

**Keywords:** calcium sulfoaluminate cement, sustainable cement, CO_2_ reduction, rapid setting concrete, shrinkage compensating concrete, ettringite

## Abstract

This study investigated the material properties and hydration characteristics of calcium sulfoaluminate cement (CSA) based mortars cured under 3 different initial curing temperatures. Two CSA cements with different M-values were selected. Obtained experimental results of mechanical properties, dimensional stability, and heat release were explained by hydration characteristics from X-ray diffraction, thermal gravimetric analysis, porosimetry, and thermodynamic modeling. Decomposition of ettringite decreased compressive strength but re-formation of ettringite after additional curing at 30 °C helped to recover the strength in CSA cement with a high amount of calcium sulfate. CSA cement with a low amount of calcium sulfate which was designed to predominantly have monosulfate as the main hydration product, showed increased 1-day strength after higher temperature curing but this occurred was at the expense of decreased 28-day strength.

## 1. Introduction

Calcium sulfoaluminate (CSA) cement was initially developed as an expansive additive for concrete [1,2]. At present, CSA cement has been considered as a sustainable alternative to replace ordinary Portland cement (OPC), since the production of the CSA cement requires less limestone and a lower calcination temperature compared to OPC [3,4,5,6]. This implies that the CSA cement is a more sustainable cement than OPC due to creating less CO_2_ emissions during its production process. In addition, CSA cement can reduce energy consumption during clinker grinding due to its more friable nature [7,8].

Ye’elimite (C_4_A_3_$\bar{S}$) is a main constituent in CSA cement and comprises approximately 30–70% of CSA cement [9]. Note that A = Al_2_O_3_, C = CaO, F = Fe_2_O_3_, H = H_2_O, M = MgO, S = SiO_2_, T = TiO_2_, $\bar{S}$ = SO_3_, and $\bar{C}$ = CO_2_ in cement chemistry notation. The ye’elimite produces monosulfate (C_4_A$\bar{S}$H_12_) and/or ettringite (C_6_A$\bar{S}$_3_H_32_) with aluminum hydroxide (AH_3_) depending on the amount of added calcium sulfate (i.e., anhydrite (C$\bar{S}$) or gypsum (C$\bar{S}$H_2_)). The calcium sulfate plays an important role in the hydration of ye’elimite [3,5,10,11,12], and the molar ratio of calcium sulfate to ye’elimite (M-value) can be utilized to categorize the properties of the CSA cements such as: (1) ‘rapid hardening and high strength’ when M-value < 1.5, (2) ‘expansive’ when 1.5 < M-value < 2.5, and (3) ‘self-stressing’ when M-value > 2.5 [12,13].

The hydration of ye’elimite is also influenced by the water-to-cement ratio (w/c) [5,14], the presence of fillers [4,15,16], and the presence of supplementary cementitious materials (SCMs) [17,18,19,20]. Several studies [5,15] reported that a low w/c caused significant self-desiccation due to the high water consumption for ettringite formation, inducing incomplete hydration of the CSA cements. Jeong et al., [5] found that strätlingite (C_2_ASH_8_) was experimentally identified by XRD only in the samples with a high w/c. Limestone filler can improve the mechanical properties and hydration of CSA cement, inducing the formation of hemicarboaluminate (C_4_A$\bar{C}$_0.5_H_10.5_) and/or monocarboaluminate (C_4_A$\bar{C}$H_11_) with the additional formation of ettringite [5,15,16], but the dilution effect by the use of the filler can be overcome when finer filler than the cement is used [4]. The incorporation of SCMs can also modify the hydration products of CSA cements, increasing the formation of strätlingite [17]. These previous studies imply that the hydration of CSA cements is highly sensitive to the cement components, admixtures, and mix design.

Ettringite, a main hydration product of CSA cement is thermally unstable at elevated temperatures [21,22,23]. The phase can decompose into meta-ettringite which losses 10–13H_2_O in the structure of ettringite [21] and can transform into monosulfate, causing delayed ettringite formation (DEF) if the ettringite is steam cured at early ages. The decomposition of the main hydration product, ettringite, can significantly influence the dimensional stability and mechanical properties of hardened CSA-based materials. On the other hand, CSA based materials could be considered for use in thermal energy storage due to its low cost, possession of high energy density, and low working temperature [24,25,26,27,28]. In construction, initial heat curing has been widely applied in the construction industry for the production of precast concrete elements [29]. Thus, the hydrothermal and mechanical properties of CSA cements cured at various temperatures have to be studied for the potential use of CSA cement in precast manufacturing [29] and thermal energy storage [26].

This study investigates the material properties and hydration behaviors of CSA cement based mortars with different M-values and varying initial curing temperatures. A series of experiments including compressive strength tests, length change measurements, isothermal conduction calorimetry, X-ray diffraction (XRD) with quantitative Rietveld refinement, and thermogravimetric (TG) analysis was carried out. Thermodynamic modeling was also conducted to interpret the complex hydration of CSA cements under different curing temperatures at an early stage.

## 2. Experimental Methods

### 2.1. Material Properties and Preparation

**Figure 1:**
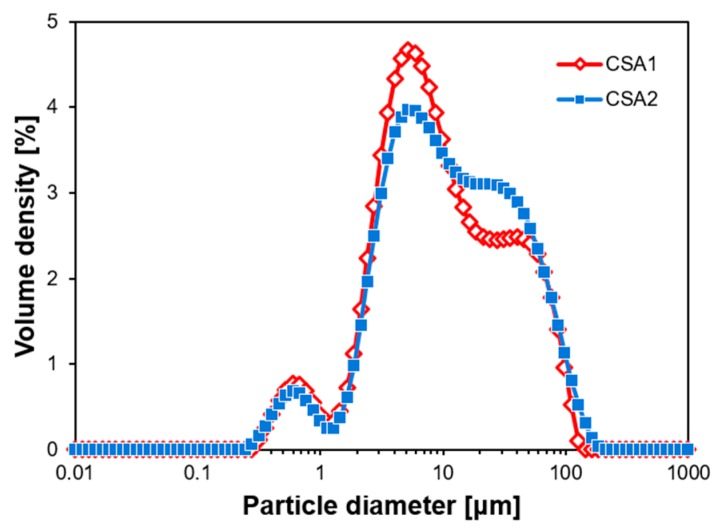
Particle size distributions of calcium sulfoaluminate (CSA) cements utilized.

Two CSA cements with different M-values were obtained by combining a micronized anhydrite with a high ye’elimite CSA clinker. Particle size distributions of the resulting two CSA cements were investigated using a laser diffraction particle size analyzer (Mastersizer 3000, Malvern Instruments Ltd., Malvern, UK) and are presented in Figure 1. The distributions of two cements are similar and fall within a range of typical CSA cements [4,5]. The oxide chemical compositions of the CSA cements were investigated using X-ray fluorescence (XRF, S8 TIGER, Bruker Co. Ltd., Karlsruhe, Germany) spectroscopy and are shown in Table 1. Note that the oxide elements lower than 0.1 wt.% are not included in Table 1.

**Figure 2:**
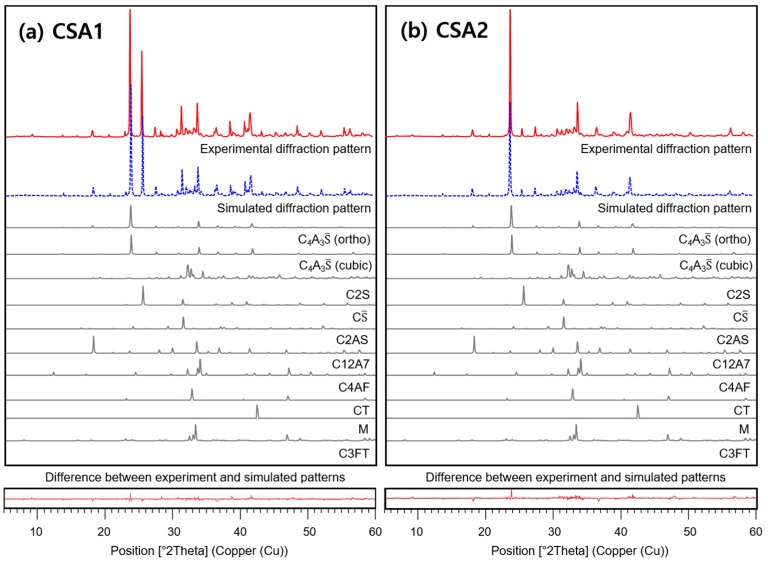
Rietveld refinement results of (**a**) CSA1 and (**b**) CSA2 cements.

The CSA cements were analyzed via X-ray diffractometer (XRD, D2 Phaser, Bruker Co. Ltd.), and Rietveld refinements were carried out to find out the mineralogical compositions of the CSA cements as shown in Figure 2. The compositions are shown in Table 1, and the results indicate that CSA1 contains more calcium sulfate than CSA2. Note that the amount of ye’elimite is the summation of both cubic and orthorhombic ye’elimite crystals. Calculated M-values of CSA1 and CSA2 are 1.86 and 0.21, respectively.

### 2.2. Experimental Procedures

The XRF, XRD, and laser diffraction particle analysis for raw CSA cements were conducted as presented in Section 2.1. Mortars were made from the two CSA cements at sand/cement (s/c) = 2.4 and water/cement (w/c) = 0.5. The mixture proportion details are presented in Table 2. Mixing procedures were guided by ASTM C305 [30]. Freshly mixed mortars were produced at room temperature and cast into 5 × 5 × 5 cm^3^ molds [31] and 2.54 × 2.54 × 28.57 cm^3^ prism molds for compressive strength tests and length change measurements [32], respectively. Cast mortars were sealed in vinyl plastic and transferred to a controlled environmental chamber at the target curing temperature. Total time for mixing, casting, and sealing was about 15 min. After the 24 h of initial curing in the chamber, samples were demolded, and cured again under water at 30 °C before specific tests. Compressive strength tests were carried out at 1 and 28 days. The mortar lengths were measured as averaged triplicate samples through 98 days of hydration and normalized to their length at 1 day after cooling to 30 °C.

An isothermal conduction calorimeter (TAM Air, TA Instruments Ltd., New Castle, DE, USA) was utilized to investigate the rate of heat release during hydration. The pastes excluding sand in Table 2 were prepared externally and placed into a glass vial and then, their isothermal conduction calories were measured for 60 h at 30 °C and 24 h at higher temperatures of 60 °C and 90 °C. Measured heat flow was normalized to each paste sample weight.

The pastes excluding sand in Table 2 were cast into Ø2.54 cm × 2.54 cm cylindrical molds for XRD, TG, and MIP experiments. The curing program for paste samples was identical to that of mortar specimens. Hardened pastes were crushed for XRD and TG tests at 1, 14, 28, 56, and 77 days and sliced into 5 × 5 × 5 mm^3^ using a diamond precision cutter (Mecatome T180, PRESI Co. Ltd., Eybens, France) for MIP experiments. Hydration at specified ages was stopped using isopropyl alcohol (IPA) and diethyl ether, followed by briefly drying at 40 °C [33,34].

Diffraction patterns of raw CSA cements and hardened pastes were collected using an X-ray diffractometer with Cu-Kα radiation (λ = 1.5418 Å) and a scanning range from 5° to 60° in 2θ. Measured XRD reflections were analyzed using HighScore Plus software (version 3.0.5, PANalytical, Almelo, The Netherlands) [35] with the Inorganic Crystal Structure Database (ICSD) [36] and the Crystallography Open Database (COD) [37]. To explore the variation of mineralogical compositions, Rietveld refinement was conducted by refining the phase scale factors, unit cell parameters, peak profile asymmetry, zero shift, and specimen displacement with manually fixed background parameters. CeO_2_ (SRM 674b, NIST, Gaithersburg, MD, USA) was used as an internal standard for XRD quantitative analysis at a weight ratio of CeO_2_:paste = 1:9.

TG experiments were carried out using a simultaneous DSC/TG system (SDT Q600, TA Instruments Ltd.) at 1, 14, 28, 56, and 77 days. The weight loss of each paste was recorded from ambient temperature to 1000 °C at a rate of 10 °C/min under a nitrogen environment. The chemically bound water content of each paste was calculated based on the weight loss at 500 °C from the TG results and utilized for the normalization of quantitative XRD results.

The pore size distribution of sliced CSA pastes at 28 days was examined using a mercury porosimeter (AutoPore IV 9500, Micromeritics Co., Norcross, GA, USA) with a pressure range from 0.10 to 61,000 psia. Obtained pore size distributions were normalized to the weight of the hardened pastes.

Thermodynamic modeling of CSA cement hydration was carried out using the Gibbs Energy Minimization Software (GEMS, version 3, Paul Scherrer Institute, Villigen, Switzerland) [38,39] with the PSI-Nagra database [40] as well as the cement-specific CEM-DATA14 database [41,42,43]. The activity coefficients of the aqueous species were computed with the extended Debye-Hückel equation with a common ion-size parameter of 3.67 Å for KOH solutions. XRD results indicated that ye’elimite and anhydrite were the only active cement components at 1 day regardless of M-value or curing temperature, so 53.6 g ye’elimite, 23.8 g anhydrite, and 50 g water were the chemical inputs into the model for CSA1 and 64.4 g ye’elimite, 3.2 g anhydrite, and 50 g water were the chemical inputs into the model for CSA2. The modeling was performed at atmospheric pressure, and the temperature was varied from 20 °C to 99 °C to determine the effect of elevated curing on the stable phase assemblages.

## 3. Experimental Results

### 3.1. Compressive Strength and Dimensional Stability

**Figure 3:**
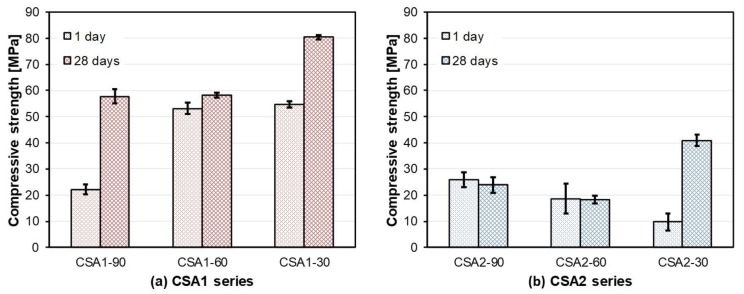
Development of compressive strength of (**a**) CSA1 and (**b**) CSA2 cement based mortars up to 28 days. Error bars of each measurement points indicate obtained standard deviations from three replicates. Specific values including its standard deviation are represented in Table S1.

The compressive strengths of CSA1 mortars were higher than CSA2 mortars at any given age or curing temperature, except CSA1-90 which had a lower strength than CSA2-90 at 1 day. (Figure 3). Firstly, the 1-day strength of CSA1 decreased by 60% by increasing the 1-day curing temperature to 90 °C; however, the 1-day strength was the same for CSA1-30 and CSA1-60. The strength of CSA1-30 & 90 significantly increased from 1 to 28 days; however, CSA1-60 showed much less strength gain over that period. Elevating the curing temperature from 30 °C to 60 °C and 60 °C to 90 °C increased the strength at 1 day for CSA2, however, there were no further strength gains from 1 to 28 days for CSA2-60 and CSA2-90. On the other hand, the strength of CSA2-30 significantly increased from 1 to 28 days. Many of these effects will be explained via XRD, TG analysis, and thermodynamic modeling in subsequent sections.

**Figure 4:**
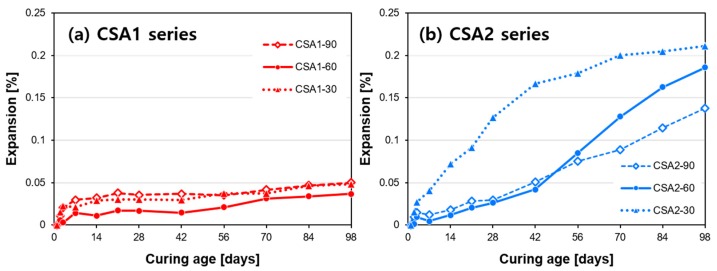
Expansion behaviors of (**a**) CSA1 and (**b**) CSA2 cement based mortars up to 98 days.

The dimensional stability of CSA mortars is presented in Figure 4. All prepared mortars show some degree of expansive behavior up through the end of measurement, 98 days. Compared to the strength results where CSA1 mortars had higher compressive strengths, CSA2 mortars were found to be considerably more expansive. CSA1 mortars show steady expansion at a faster rate through 7 days. In the case of CSA2 mortars, the expansion of CSA2-30 is greater than samples cured at elevated temperatures. However, this trend is likely to reverse beyond 98 days since the rates of expansion of CSA2-60&90 are greater than CSA2-30 from 56 to 98 days. At 98 days, CSA2 mortars have increasing total expansion with decreasing 1-day curing temperatures. In other words, at 98 days, the total measured expansion decreases in the order of CSA2-30 > CSA2-60 > CSA2-90.

### 3.2. Pore Structure

**Figure 5:**
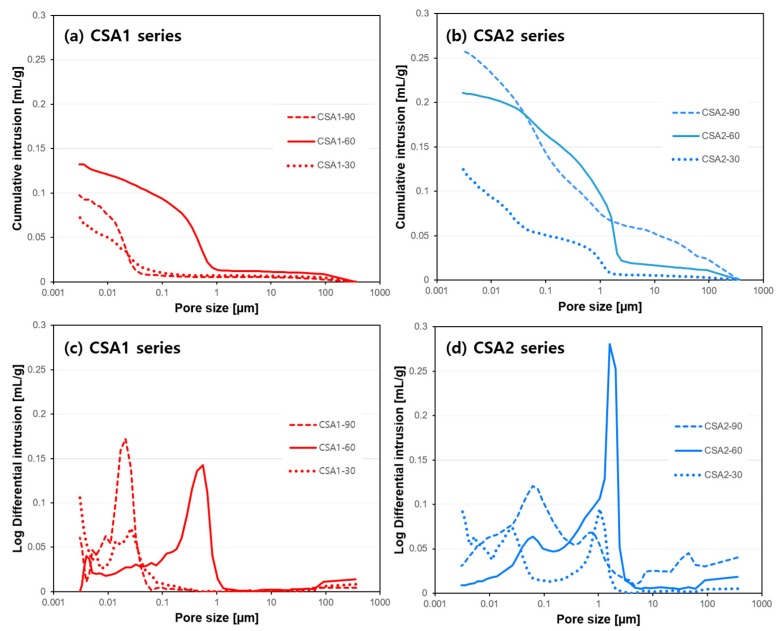
Results of mercury intrusion porosimetry (MIP) at 28 days: cumulative intrusion of (**a**) CSA1 and (**b**) CSA2 series and log differential intrusion of (**c**) CSA1 and (**d**) CSA2 series.

The results of the pore structure analysis on CSA pastes at 28 days of curing are shown in Figure 5. In general, more porosity and coarser pores were found in CSA2 pastes regardless of the curing temperature, which contribute to CSA2’s lower compressive strengths (Figure 3). Both CSA1 and CSA2 pastes cured at 30 °C had smaller pore volumes compared to those cured at elevated temperatures. At elevated 1-day curing temperatures, increasing from 60 °C to 90 °C caused the pores to become finer as evidenced by the dominate pore size decreasing from around 1 µm to less than 0.1 µm for both CSA1 and CSA2. However, the cumulative pore volume was decreased in CSA1 and increased in CSA2 mortars due to increasing the 1-day curing temperature from 60 °C to 90 °C.

### 3.3. Heat of Hydration

**Figure 6:**
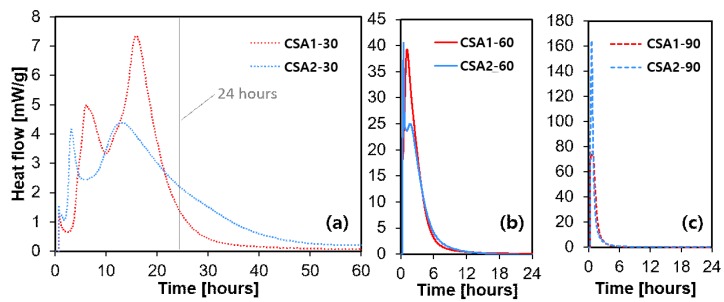
Isothermal conduction calorimetry of each samples cured at (**a**) 30 °C, (**b**) 60 °C and (**c**) 90 °C.

**Figure 7:**
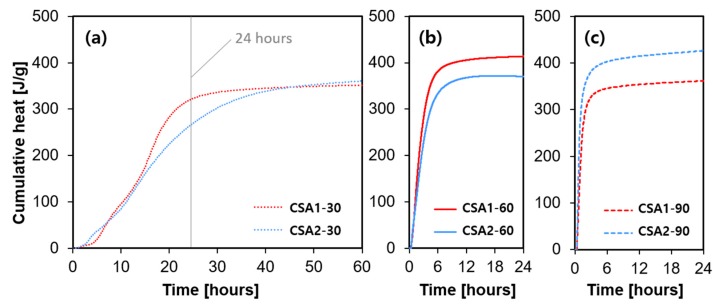
Cumulative heat releases of each samples cured at (**a**) 30 °C, (**b**) 60 °C and (**c**) 90 °C.

The heat flow evolution of CSA pastes is shown in Figure 6. Note that the ordinate scale changes in Figure 6 with changing curing temperature. At the curing temperature of 30 °C, two main peaks were observed. The two peaks initiated earlier in the CSA2-30 sample, but the peaks of CSA1-30 have higher intensity than those of CSA2-30. This is due to the acceleratory effect of calcium sulfate on ye’elimite hydration. In general, as the curing temperature rose, the maximum rate of heat flow (i.e., the height of the main hydration peak) significantly increased and occurred earlier. It is interesting to note that CSA1-60 emits more heat than CSA2-60, while the opposite occurs at the curing temperature of 90 °C, as shown in Figure 7b,c. Moreover, the heat released at 24 h by CSA2 increased with an increasing curing temperature. However, the heat released at 24 h by CSA1 increased from 30 °C to 60 °C, but decreased from 60 °C to 90 °C. This might be explained by the endothermic reaction of the decomposition of ettringite at higher temperatures [24,25], considering the condition (i.e., external mixing) of the conduction calorimetry measurement. This phenomenon also explains the lower compressive strength of CSA1 at 1 day with a curing temperature of 60 °C compared to 90 °C.

### 3.4. X-ray Diffraction and Thermogravimetric Analysis

**Figure 8:**
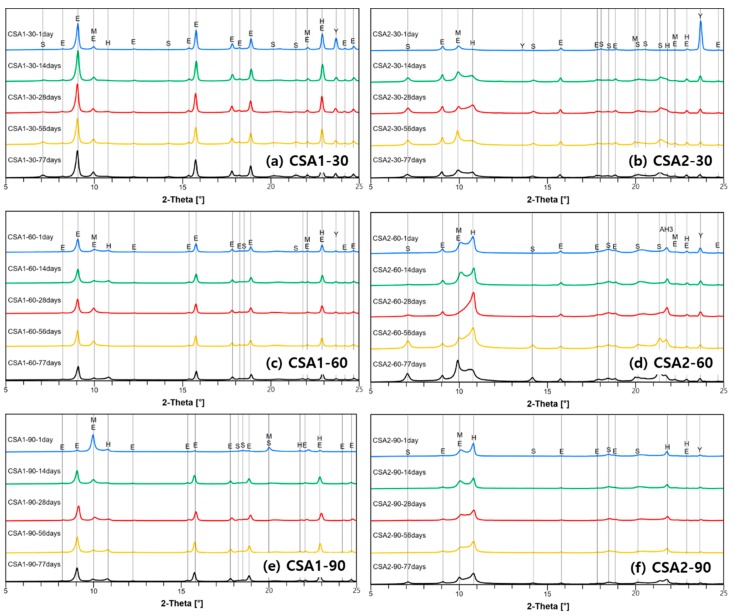
Measured X-ray diffraction patterns of each samples cured at (**a**,**b**) 30 °C, (**c**,**d**) 60 °C, and (**e**,**f**) 90 °C. Symbols of E, M, H, S, and Y indicate ettringite, monosulfate, hemicarboaluminate, and ye’elimite, respectively.

The results of XRD analysis of the hydrated CSA pastes at different curing ages of 1, 14, 28, 56, and 77 days are shown in Figure 8. In the figure, a 2 theta range of 5° to 25° is presented to highlight the main hydration phases. In addition, quantitative refinement results for curing ages are included in the Supplementary Figure S1. Clearly, the CSA1 pastes have more ettringite than CSA2 pastes due to the existence of more anhydrite in the cement (Table 1). Nevertheless, the CSA2-30 and CSA2-60 had a small amount of ettringite at all curing ages, whereas there is no ettringite found in CSA2-90 at 1 day. It was experimentally confirmed that almost complete ettringite destabilization and decomposition occurred at a curing temperature of 90 °C for both CSA cements, as expected. In CSA1-90 and CSA2-90, the decreased ettringite reappeared at 14 days of curing, but the peak intensity did not change after that. This reappearance of ettringite was more evident in CSA1-90 but was weakly observed in CSA2-90. More monosulfate and less ettringite are produced in the lower M-value CSA2 pastes; moreover, more strätlingite is produced in CSA2, which is consistent with prior research [5]. Finally, increasing the 1-day curing temperature increases the amount of solid solution AFm phases produced in both CSA1 and CSA2 pastes but to a greater extent in the lower M-value (CSA2) pastes.

**Figure 9:**
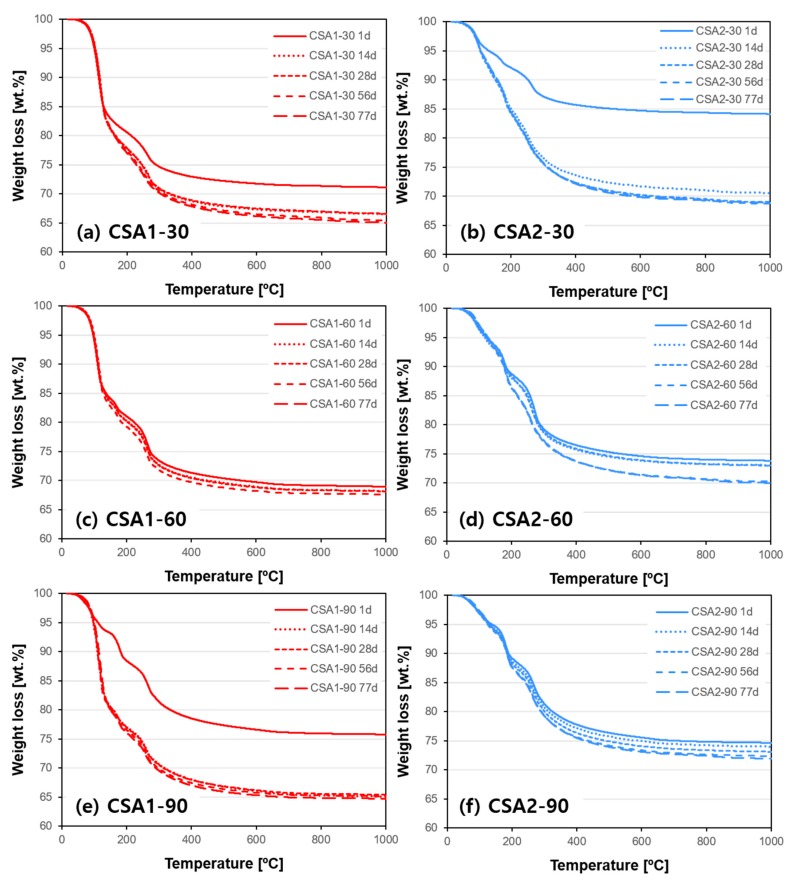
Weight loss curves obtained from thermogravimetric analysis (TGA) of each samples cured at (**a**,**b**) 30 °C, (**c**,**d**) 60 °C, and (**e**,**f**) 90 °C.

**Figure 10:**
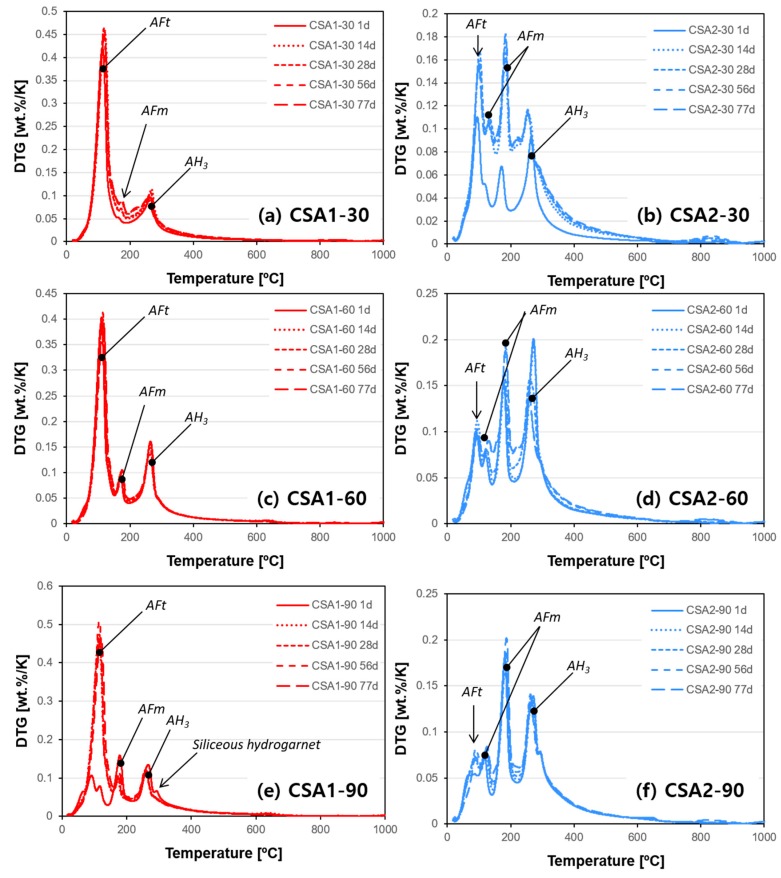
Derivative weight loss curves from TGA of each samples cured at (**a**,**b**) 30 °C, (**c**,**d**) 60 °C, and (**e**,**f**) 90 °C.

The TGA and DTG results are presented in Figure 9 and Figure 10, respectively. Hydration stopped paste samples with identical curing ages used for XRD were measured. Overall, the weight loss increases with curing age in all paste samples but detail trends are quite different depending on the type of CSA cement and curing temperature. In the cases of CSA1-60, CSA2-60, and CSA2-90, relatively small increases in weight losses over time in Figure 9c,d,f explain the stable compressive strength of them from 1 to 28 days (Figure 3), since this is indicative of low hydration activity. On the other hand, CSA1-30, CSA1-90, and CSA2-30 present significant gains of compressive strength from 1 to 28 days (Figure 3). The strength gain in theses samples can be explained by the additional hydration reactions occurring over time confirmed in Figure 9a,b,e for CSA1-30, CSA1-90, and CSA2-30, respectively. Similar trends with more information on hydration phases can be confirmed in the DTG data (Figure 10). In agreement with the XRD results, the DTG analysis shows less ettringite (AFt) and more AFm type phases forming in the lower M-value CSA2 pastes. Finally, when curing at 90 °C a siliceous hydrogarnet peak can be observed in the DTG results for CSA1 and CSA2. This result is in agreement with Dilnesa et al., who observed that siliceous hydrogarnet formed under hydrothermal curing conditions [44]. Although XRD results did not indicate the dissolution of C_2_S at 1 day, either a small amount (below XRD sensitivity) of C_2_S may have dissolved or the silica could have come from the ye’elimite dissolution, since ye’elimite can accommodate some substitution [45]. A more comprehensive discussion on this observation will be followed with XRD and GEMS interpretations in the Discussion section.

### 3.5. Thermodynamic Modeling

**Figure 11:**
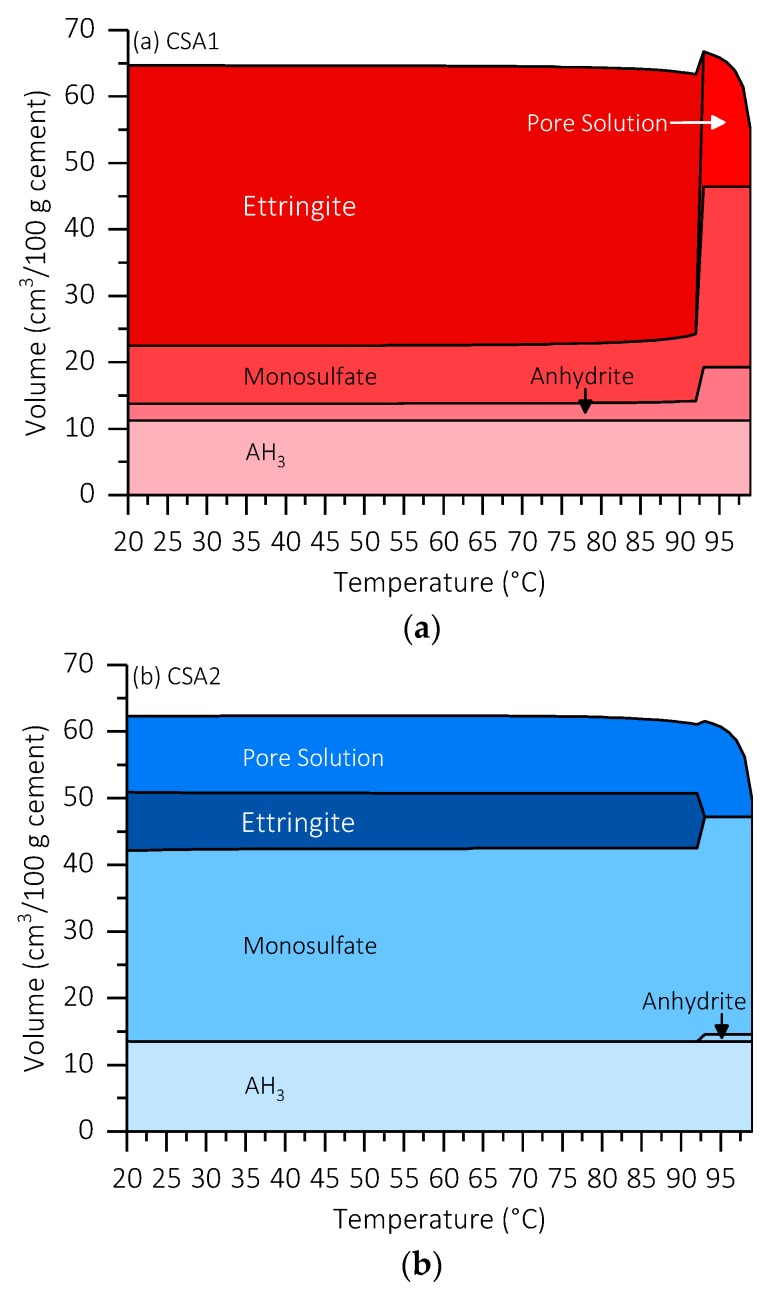
Thermodynamic modeling showing effect of elevated curing temperature on ye’elimite and anhydrite hydration with a w/c = 0.5 for (**a**) CSA1 and (**b**) CSA2.

To better understand the effect of increasing the curing temperature on the hydration of CSA1 and CSA2, the active cement phases at 1 day (ye’elimite and anhydrite) from Table 1 were combined with 50 g water and the temperature was varied from 20 °C to 99 °C. Figure 11 presents the results of the modeling. In the modeling at approximately 93 °C, ettringite is destabilized and the remaining stable phases are monosulfate, AH_3_, and anhydrite, although experimentally the temperature at which ettringite destabilizes can be lower. It is interesting to note that anhydrite is the stable form of calcium sulfate in contact with water at this temperature and not gypsum. Actually, when considering elevated curing, anhydrite becomes the stable form of calcium sulfate at approximately 48 °C, but this temperature can vary depending on the pore solution and its concentration [46]. The stability of anhydrite at elevated temperatures helps explain the additional 10 g of anhydrite present in CSA1-90 compared to CSA1-30 after 1 day of curing. This raises the question as to whether high M-value (~2) high ye’elimite cements would receive early age strength gain benefits by utilizing gypsum instead of anhydrite while curing at a moderately elevated temperature well below the ettringite destabilization temperature (i.e., 60 °C).

## 4. Discussion

### 4.1. Hydration Characteristics at 30 °C Curing Temperature

CSA cements typically react faster than OPC, owing to the rapid hydration of ye’elimite [47,48]. As previous studies have shown, incorporation of calcium sulfate in CSA cement improves the compressive strengths of the mortars, and the volumetric expansion from ettringite formation is known to be controllable [49]. Ye’elimite’s reaction generally follows two chemical equations (Equations (1) and (2)) depending on the available amount of calcium sulfate.

(1) $$C_{4}A_{3}\bar{S}+2C\bar{S}H_{2}+34H\to C_{3}A\cdot 3C\bar{S}\cdot H_{32}{+2\mathrm{AH}}_{3}$$

(2) $$C_{4}A_{3}\bar{S}+18H\to C_{3}A\cdot C\bar{S}\cdot H_{12}{+2\mathrm{AH}}_{3}$$

When curing at 30 °C, strätlingite was found more abundantly and more unreacted ye’elimite was present (Figure 8). Due to the low amount of anhydrite in CSA2, there was less formation of ettringite but more formation of monosulfate and hemicarboaluminate. Strätlingite is known to form from belite as a Si and Ca source and ye’elimite as a Ca and Al source, or aluminum hydroxide as an Al source [10,50]. The main hydration reaction for strätlingite formation from belite and aluminum hydroxide is (Equation (3))

C_2_S + AH_3_ + 5H → C_2_ASH_8_ (3)

As confirmed by TGA and DTG (Figure 9 and Figure 10), continued hydration reactions binding more water from 1 to 28 days leads to additional development of compressive strength at 28 days (Figure 3). While there is not much difference in the expansion behavior of the CSA1 series, CSA2 displayed a faster initial rate of expansion when continuously cured at 30 °C and an increased rate of expansion at later ages when cured for 1 day at elevated temperatures. Beyond 28 days, CSA2 mortars showed greater amounts of expansion than CSA1 mortars, regardless of the initial curing temperature. Many studies have shown that expansion generally increases with increasing calcium sulfate in CSA cements due to the increased production of ettringite [3,7], so CSA2 with its lower M-value expanding more than CSA1 might seem unusual. However in 2014, Hargis et al. [51] observed 0.8% expansion in a ye’elimite paste without added calcium sulfate at 84 days of hydration; moreover, Hargis et al. [52] building on the work of other research [53,54] investigating the role of crystallization pressure on cement expansion showed that in addition to ettringite other phases such as monosulfate, strätlingite, and CAH_10_ could contribute to the total crystallization pressure exerting expansive forces in CSA cement pastes.

### 4.2. Decomposition and Re-Formation of Ettringite

Ettringite experiences decomposition at elevated temperatures and re-formation after cooling and allowing time for ettringite to recrystallize (also known as delayed ettringite formation) [21,22,23]. Ettringite can thermally decompose into meta-ettringite [21] and can transform into monosulfate and basanite (Equation (4)) or into hydrogarnet (Equation (5)) [55,56,57]. Siliceous hydrogarnet was observed in CSA1-90 and CSA2-90.

(4) $$C_{3}A\cdot 3C\bar{S}\cdot H_{32}\to C_{3}A\cdot C\bar{S}\cdot H_{12}+2C\bar{S}H_{0.5}+19H$$

(5) $$C_{3}A\cdot 3C\bar{S}\cdot H_{32}\to C_{3}{\mathrm{AH}}_{6}+3C\bar{S}+26H$$

In the current study, complete and partial decompositions of ettringite was observed in samples cured in 90 °C and 60 °C, respectively. While there is not much change in CSA1-60, the decomposed ettringite reappeared in CSA1-90 by 14 days. For the CSA2 series, less ettringite initially formed owing to the lower amount of anhydrite in the cement. Instead, larger amounts of monosulfate and hemicarboaluminate were observed (Equation (2)). Similarly, in CSA2-90, less ettringite reformed after the initial destabilization due to the elevated curing period, compared to CSA1-90. Another observation is that there was very little change in the bound water in CSA1-60 and CSA1-90 after 14 days of curing, indicating slight changes in the hydration products. Whereas, there were larger increases in the bound water and increases in strätlingite formation in CSA2-60 and CSA2-90 (Equation (3)). However, compared to the previous finding of strength gain by strätlingite formation [5], the strätlingite formation or aluminum hydroxide consumption did not help to increase the compressive strength for CSA2-60 and CSA2-90 from 1 to 28 days.

With 1-day curing at 90 °C, CSA2 emits more heat than CSA1 whereas it is the opposite when curing at 60 °C (Figure 6 and Figure 7). This is probably due to the effects of ettringite decomposition/destabilization and reduced anhydrite dissolution outweighing the additional heat released by more ye’elimite dissolution in CSA1 [25,26]. Since CSA2 produces less ettringite and more monosulfate and other AFm-type phases, increasing the 1-day curing temperature causes more reaction of the anhydrous phases with less of the negative side effects of ettringite destabilization. This can help explain the 1-day strength enhancement of the CSA2 series by raising the curing temperature from 30 to 60 or 90 °C; whereas, in CSA1-90, the decomposition and destabilization of ettringite lead to a reduced strength at 1 day (i.e., 60% of strength reduction). In short, raising the curing temperature caused the 1-day strength of CSA2 to increase due to the formation of more AFm-type hydration products but decreased the strength in CSA1 by decreasing the amount of ettringite formed. Therefore, it can be concluded that raising the curing temperature for high ye’elimite CSA cements with sufficient added calcium sulfate to produce ettringite as the main hydration product is not beneficial. It led to low strength at 1 day, and the regaining of strength due to the re-formation of ettringite did not reach the 28-day strength level achieved by continuously curing at 30 °C. Additionally, it could expose the cement to the potential of delayed ettringite formation causing deleterious expansion at later ages than observed in this study. In contrast, the hydration reaction of CSA cement with low amounts of calcium sulfate can be accelerated by elevated temperature curing. This rapid hydration at elevated temperature consumes ye’elimite quickly; thus, it makes further hydration less active. This eventually led to lower strength at 28 days compared to CSA cement cured continuously at 30 °C, which experienced more extended hydration reactions. The formation of strätlingite was increased with elevated curing temperatures in CSA2. Although its effect on the strength development looks negligible, it in combination with other hydration products forming may contribute to the increased rate of expansion at later age of curing ages.

In regard to expansion, elevated temperature curing caused delayed expansion for CSA2 mortars (Figure 4). Similar with the case of CSA1, increasing temperature to 60 °C decreased the amount of ettringite and to 90 °C completely destabilized the ettringite. By 14 days of curing, the ettringite had begun to re-form in CSA2-60 and CSA2-90 but it was not the same extent as in CSA1-60 and CSA1-90. The continued expansion though 98 days (Figure 4) suggests that additional expansion can still occur beyond the final measurement date of this study. This could be particularly problematic in CSA1, which had more ettringite destabilized by elevated curing and has yet to fully reform at 77 days as evidenced by the lower intensities of the ettringite peaks for CSA1-60 and CSA1-90 compared to CSA1-30.

### 4.3. Relations of Material Properties with Hydration Characteristics

**Figure 12:**
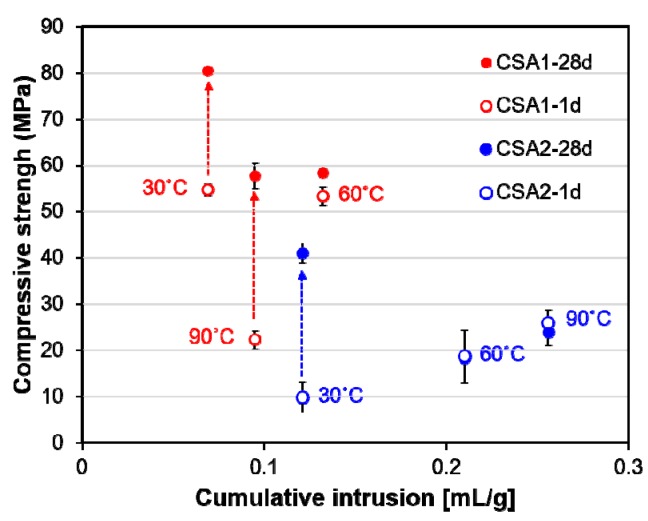
Relationship between compressive strength measured at 1 day and 28 days and cumulative intrusion data measured at 28 days. Grey arrows indicate the gain of strength from 1 day to 28 days for selected samples. Note that two points of strength of CSA2-60 at 1 and 28 days are overlapped.

**Figure 13:**
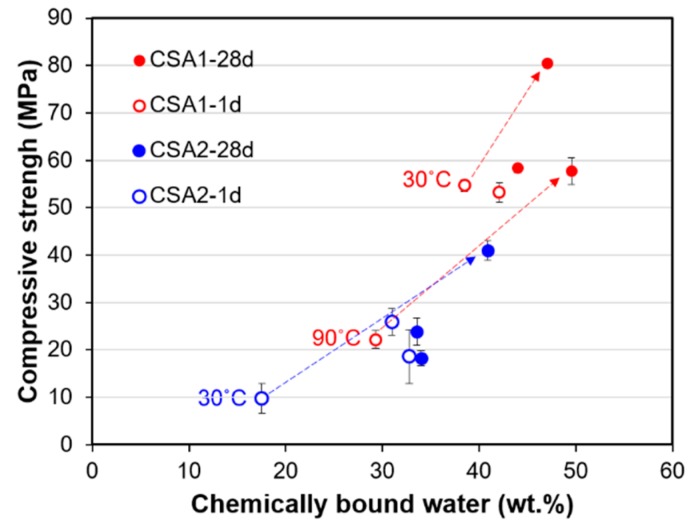
Inverse relationship between compressive strength measured at 1 day and 28 days and chemically bound water contents obtained by TGA on each samples cured at 1 day and 28 days. Grey arrows indicate the enhancement of strength from 1 day to 28 days and corresponding increase of chemically bound water contents from 1 day to 28 days.

Figure 12 presents the observed inverse relationship between compressive strength at 28 days (filled circle in the figure) and cumulative intrusion measured at 28 days. Regardless of curing temperature, generally large pores in CSA2 led to lower strength. In addition, the lower pore volumes in CSA1 and CSA2 cured at 30 °C yields the highest strengths at 28 days. Considered together with TGA results, there is also a clear relationship between the compressive strengths and the amount of chemically bound water (Figure 13). In other words, the degree of hydration has a direct influence on the compressive strength at 1 and 28 days. Compared to the CSA2 series, the CSA1 series with more anhydrite contained more chemically bound water, including the amount from the re-formation of ettringite after the elevated curing at 1 day. These large increases in the degrees of hydration (as evidenced by the large increases in the TG weight losses) between 1 and 28 days in CSA1-30, CSA1-90, and CSA2-30 clearly contributed to the significant strength gains in those samples (indicated as grey arrows in Figure 12 and Figure 13).

## 5. Conclusions

For calcium sulfoaluminate (CSA) mortars with a high amount of calcium sulfate (M-value of 1.86), elevated curing temperature at or above 60 °C for 1 day was not helpful for CSA cement formulations predominately designed to produce ettringite as a hydration product. Due to the decomposition of ettringite, the strength of CSA mortars cured at 90 °C was lower than those cured at 30 °C and 60 °C for 1 day. The effect of elevated curing on expansive behavior was negligible over 98 days; however, the potential for future expansion due to delayed ettringite formation exists because the same amount of ettringite had not re-formed at 77 days as was produced in the paste cured continuously at 30 °C. Compared to 1-day curing at 60 °C, there was significant strength gain from 1 to 28 days when curing for 1 day at 90 °C, which was explained by the re-formation of ettringite. However, this delayed strength gain was still lower than that obtained at 28 days by curing at 30 °C continuously.

On the other hand, elevated curing at or above 60 °C was useful to increase the 1-day strength in CSA cement formulations designed to mainly produce monosulfate (M-value of 0.21). However, the increased 1-day strength came at the expense of strength at 28 days. Due to the lack of sulfate supply, there was limited formation of ettringite both during and after the elevated temperature curing. The mortar cured continuously at 30 °C expanded more than the mortars cured at elevated temperatures for 1 day, especially before 42 days; whereas, the rate of expansion for samples which experienced an elevated curing temperature for 1 day increased after 56 days.

Regardless of calcium sulfate content, there was an inverse relationship between compressive strength at 28 days and cumulative pore volume measured at 28 days. Also, for both high and low M-value CSA mortars, the increase in compressive strength between 1 and 28 days was correlated to an increase in the amount of chemically bound water (the degree of hydration).

Results reported herein deepen the understanding on mechanical and dimensional stability of CSA-based materials for thermal energy storage applications and for precast companies considering steam curing CSA structural elements.

## Figures and Tables

**Table 1:** Chemical and mineralogical compositions of CSA cements.

XRF			QXRD		
Oxide	CSA1 (wt.%)	CSA2 (wt.%)	Phases	CSA1 (wt.%)	CSA2 (wt.%)
CaO	41.3	41.3	C_4_A_3_$\bar{S}$ (ortho)	40.0	51.0
Al_2_O_3_	28.1	34.5	C_4_A_3_$\bar{S}$ (cubic)	13.6	13.4
SiO_2_	5.6	6.96	β-C_2_S	9.7	15.0
SO_3_	18.0	9.46	C$\bar{S}$	23.8	3.2
Fe_2_O_3_	1.6	2.0	C_2_AS	1.6	4.0
TiO_2_	1.2	1.4	C_12_A_7_	1.7	2.1
MgO	1.1	1.4	C_4_AF	0.9	1.0
Br	1.4	1.4	CT	1.1	1.4
K_2_O	0.2	0.2	M	0.6	0.7
SrO	0.1	0.1	C3FT	6.8	8.1
ZrO_2_	0.1	0.1	-		
LOI ^†^	1.2	1.0			

^†^ Loss on ignition.

**Table 2:** Mixture proportions in weight ratios (g).

Label	CSA Cement		Sand	Water	Curing Condition
	CSA1	CSA2			
CSA1-30	100	-	240	50	Cured at 30 °C for all times
CSA2-30	-	100	240	50	
CSA1-60	100	-	240	50	Cured at 60 °C for 24 h then cured at 30 °C until testing
CSA2-60	-	100	240	50	
CSA1-90	100	-	240	50	Cured at 90 °C for 24 h then cured at 30 °C until testing
CSA2-90	-	100	240	50	

## Supplementary Materials

The following are available online at https://www.mdpi.com/1996-1944/12/7/1072/s1, Table S1: Averaged compressive strength and its standard deviation, Figure S1: QXRD results for (a) CSA1-30, (b) CSA2-30, (c) CSA1-60, (d) CSA2-60, (e) CSA1-90, and (f) CSA2-90.
