# Supplementary material for: The Effect of Elevated Curing Temperatures on High Ye’elimite Calcium Sulfoaluminate Cement Mortars
Source: Materials (Basel). 2019 Apr 1;12(7):1072. doi: 10.3390/ma12071072 (PMC6480238; doi:10.3390/ma12071072)
Supplement: Supplementary file 1 [file materials-12-01072-s001.pdf]

materials

Supplementary Materials: The Effect of Elevated Curing Temperatures on High Ye’elimite Calcium Sulfoaluminate Cement Mortars

Yeonung Jeong, Craig W. Hargis, Hyunuk Kang, Sung-Chul Chun and Juhyuk Moon

Table 1. Averaged compressive strength and its standard deviation.

Sample label	Curing Age (days)	Compressive Strength (MPa)	
		Averaged Value	Standard Deviation
CSA1-90	1	22.20	1.94
	28	57.73	2.73
CSA2-90	1	25.93	2.82
	28	23.91	2.91
CSA1-60	1	53.20	2.04
	28	58.33	0.93
CSA2-60	1	18.61	5.69
	28	18.29	1.62
CSA1-30	1	54.72	1.22
	28	80.39	0.87
CSA2-30	1	9.78	3.18
	28	40.97	2.11

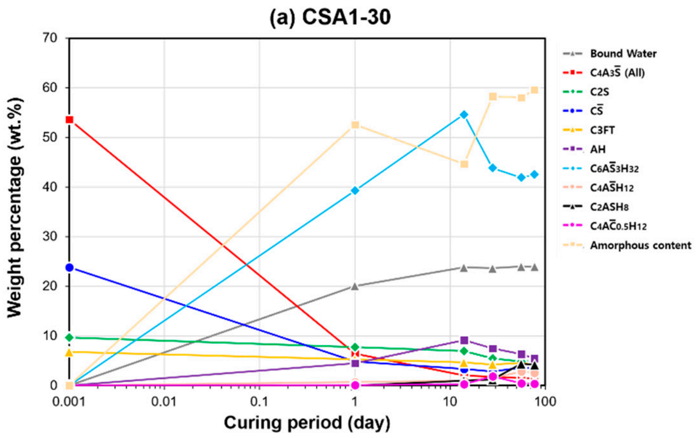

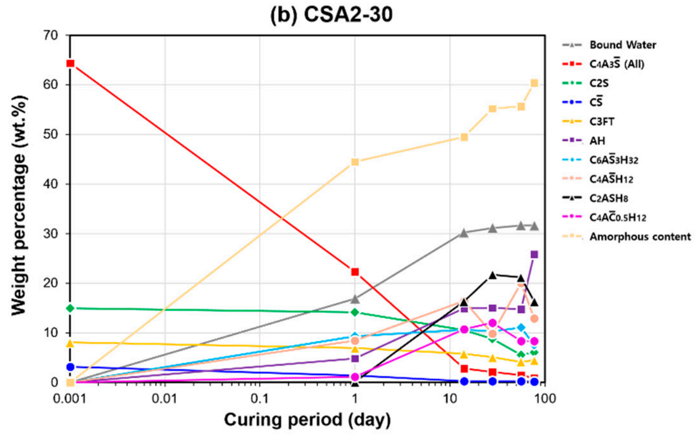

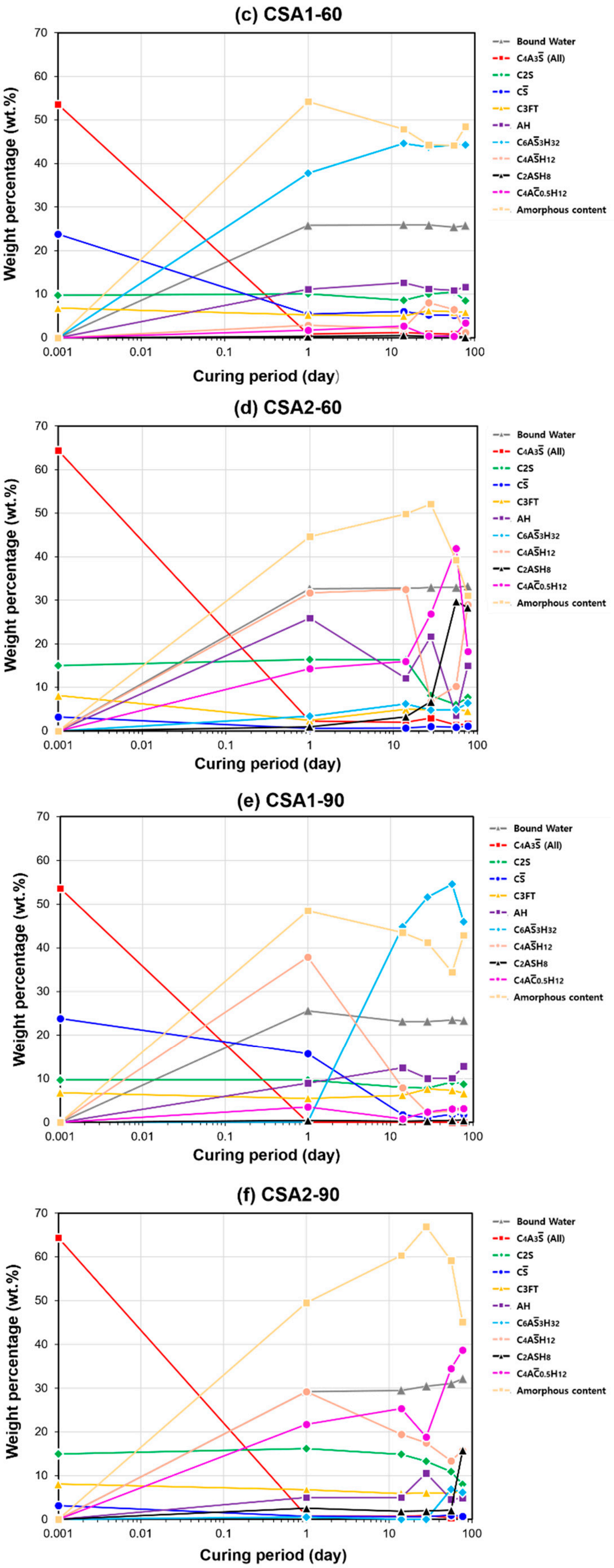

Figure S1. QXRD results for (**a**) CSA1-30, (**b**) CSA2-30, (**c**) CSA1-60, (**d**) CSA2-60, (**e**) CSA1-90, and (**f**) CSA2-90.

© 2019 by the authors. Submitted for possible open access publication under the terms and conditions of the Creative Commons Attribution (CC BY) license (http://creativecommons.org/licenses/by/4.0/).
